# Detection of Salivary Helicobacter pylori in Pediatric Patients and Its Association With Gastrointestinal and Oral Health

**DOI:** 10.7759/cureus.100744

**Published:** 2026-01-04

**Authors:** Rojin Samani, Mehrzad Sadredinamin, Naghi Dara, Ali Asghar Soleymani, Mojdeh Hakemi-Vala

**Affiliations:** 1 Dentistry, Shahid Beheshti University of Medical Sciences, Tehran, IRN; 2 Microbiology, Shahid Beheshti University of Medical Sciences, Tehran, IRN; 3 Pediatric Gastroenterology, Shahid Beheshti University of Medical Sciences, Tehran, IRN; 4 Pediatric Dentistry, Shahid Beheshti University of Medical Sciences, Tehran, IRN

**Keywords:** caga, dental caries, dmft/dmft index, endoscopy, helicobacter pylori, oral health, pediatric, polymerase chain reaction (pcr), saba, saliva

## Abstract

Background

*Helicobacter pylori *(*H. pylori*) infection is one of the most common bacterial infections worldwide and is recognized as a major cause of gastritis. The oral cavity has been suggested as a potential reservoir for this bacterium. Yet, the relationship between its presence in the mouth and stomach, as well as its possible role in dental caries, remains unclear. This study aimed to investigate the prevalence of *H. pylori* contamination in the saliva of patients aged four to 18 years undergoing endoscopy.

Materials and methods

This cross-sectional study included 100 children aged four to 18 years who underwent endoscopy due to gastrointestinal symptoms. Saliva samples were collected before endoscopy, and gastric biopsies were analyzed for *H. pylori* using standard histopathological methods, specifically the Giemsa stain. The presence of *H. pylori* was confirmed through pathology results. In addition, polymerase chain reaction (PCR) targeting the 23S rRNA gene was used to detect the *H. pylori* in saliva, and the virulence genes sabA and cagA were also examined. Dental status was assessed using the Decayed/decayed, Missing/missing, and Filled/filled Teeth/teeth (DMFT/dmft) score according to World Health Organization (WHO) criteria. Data were analyzed using descriptive and inferential statistics, including t-tests, correlation analyses, and logistic regression; p<0.05 was considered significant.

Results

Overall, 61 patients (61%) tested positive for *H. pylori* in gastric biopsy specimens. In saliva samples, 53 patients (53%) were positive for *H. pylori*. Notably, all saliva-positive patients were also positive in biopsy specimens, indicating complete overlap. Among the saliva-positive samples, the virulence gene cagA was detected in eight samples (8%). In contrast, the sabA gene wasn't detected in any sample. Notably, children with *H. pylori*-positive saliva had a significantly higher mean number of dental caries than saliva-negative children (4.10 vs. 2.05, p<0.001).

Conclusion

Saliva can serve as a reservoir for *H. pylori* in Iranian children and could be associated with dental caries severity. Saliva testing may provide a simple, non-invasive method for early detection of *H. pylori*, while promoting oral hygiene and regular dental care can help prevent recurrent gastric infections and improve overall health of the children.

## Introduction

*Helicobacter pylori *(*H. pylori*) is a Gram-negative, microaerophilic, spiral-shaped bacterium that colonizes the gastric mucosa and is recognized as a major cause of gastrointestinal diseases such as chronic gastritis, peptic ulcer, and gastric carcinoma [1,2]. According to the World Gastroenterology Organization (2021), approximately half of the global population is infected with *H. pylori*, with marked variation across geographic regions, age, ethnicity, and socioeconomic status [3]. Rates range from less than 30% in some developed countries to as high as 80% in low- and middle-income regions, reflecting differences in hygiene and living conditions [4]. In Iran, available evidence indicates that approximately 62% of adults and 42% of children are infected, reflecting a considerable public health burden across age groups [5].

Transmission of *H. pylori *occurs mainly through oral-oral and fecal-oral routes, with person-to-person contact, contaminated food, and unsafe water playing significant roles. Once established in the gastric mucosa, the bacterium can persist for years [6]. Current treatment typically involves a combination of systemic antibiotics and proton pump inhibitors; however, reinfection and treatment failure remain common. This persistence suggests the involvement of non-gastric reservoirs that are not fully affected by systemic antibiotic therapy, such as dental biofilm, saliva, and periodontal tissues. The oral cavity provides favorable ecological conditions - such as dental biofilm, suitable pH, and microaerophilic niches - that may enable bacterial colonization. Low antibiotic concentrations in the oral cavity and dental plaque may allow bacteria to survive despite gastric eradication therapy, making the mouth a potential reservoir for reinfection [7]. Indeed, *H. pylori* has been detected in dental plaque and saliva, particularly in individuals with poor oral hygiene or dental caries. Although its presence has been linked to various oral conditions, including halitosis, glossitis, recurrent aphthous stomatitis, and dental caries in some cases, there is still controversy surrounding these associations [8-10].

Since a *H. pylori* infection is typically acquired in the childhood, identifying potential oral reservoirs is essential to improve prevention, treatment efficacy, and long-term disease control. Despite global interest in the oral-gastric relationship of *H. pylori*, data from Iran remain limited, particularly among pediatric populations. Therefore, this study aimed to: (1) Determine the prevalence of *H. pylori *in saliva among children and adolescents aged four to 18 years undergoing endoscopy at Mofid Children's Hospital, Tehran, Iran, in 2023; (2) Assess the association between the presence of *H. pylori *in saliva and oral health status, particularly dental caries; (3) Evaluate the diagnostic performance of salivary polymerase chain reaction (PCR) for detecting a *H. pylori* infection; and (4) Investigate the prevalence of *H. pylori* virulence genes in saliva.

## Materials and methods

Study design and participants

This cross-sectional, observational study was conducted at Mofid Children's Hospital, Tehran, Iran, in 2023, focusing on children aged four to 18 years who were referred for endoscopy due to gastrointestinal symptoms. A total of 100 children participated in the study. The inclusion criteria were children with gastrointestinal symptoms who required endoscopic examination, while the exclusion criteria included antibiotic use or antibacterial mouthwash within the past four weeks, and any severe systemic disease or immune deficiency. The study was approved by the Institutional Review Board of the Shahid Beheshti Medical University, Tehran, Iran (Approval Number: IR.SBMU.DRC.REC.1403.082), and informed consent was obtained from the parents or legal guardians of all participants.

Sample size was estimated using the following formula: $n = \frac{z_{\frac{1-\alpha}{2}}^2 \times p(1 - p)}{d^2}$, where Z=1.96 (for a 95% confidence level), p=0.6 (estimated prevalence based on pre-study data), and d=0.1 (margin of error). The calculation resulted in a sample size of 92.19, which was rounded to 100 participants.

Data collection

A structured questionnaire was used to gather demographic data, lifestyle habits, and oral hygiene practices, including dental visits and toothbrushing frequency. Clinical dental examinations assessed the Decayed, Missing, and Filled Teeth (DMFT/dmft) index according to the World Health Organization's (WHO) oral health standards [11].

Sample collection and processing

Saliva samples were collected before undergoing endoscopy to avoid interference. The average saliva volume collected from each participant was approximately 3 mL, which was stored in sterile containers and transported at room temperature to the microbiology laboratory at the Shahid Beheshti University. Samples were then stored at 4°C until DNA extraction to preserve DNA integrity.

Detection of *H. pylori* and associated virulence genes in the saliva

*H. pylori* presence in gastric biopsy samples was analyzed using histopathological methods, specifically the Giemsa stain, following standard pathology protocols. The presence of *H. pylori* was confirmed based on the pathology results. In the saliva samples, *H. pylori* was detected by PCR targeting the 23S rRNA gene, and virulence genes cagA and sabA were also examined.

In this study, salivary PCR was selected for *H. pylori* detection due to its significant advantages over traditional culture methods. While gastric biopsy culture is considered the gold standard, it requires stringent microaerophilic conditions that are difficult to maintain, as well as specialized equipment, making it both time-consuming and labor-intensive. In contrast, PCR is a rapid, cost-effective, and widely accessible method capable of detecting *H. pylori* DNA even from dead bacteria, which is particularly useful in clinical settings where bacterial viability may be compromised during sample transport or storage. PCR provides a non-invasive, efficient alternative to gastric biopsy, delivering accurate results even when bacterial viability is uncertain. Additionally, PCR can detect *H. pylori* virulence genes, such as cagA and sabA, which are not always identified through culture techniques.

DNA was extracted from saliva using the Sambio DNA extraction kit (Sambio^TM^, South Korea). The quality of the extracted DNA was evaluated using a NanoDrop spectrophotometer (Thermo Fisher Scientific, USA). DNA samples with an A260/A280 ratio between 1.8 and 2.0 were considered of acceptable quality for PCR analysis.

PCR amplification targeted the 23S rRNA gene of *H. pylori* and virulence genes (cagA, sabA) using specific primers (Metabion, Germany) (Table 1).

**Table 1:** Primer sequences, product sizes, and annealing temperatures for H. pylori detection and associated virulence genes in saliva samples

Gene	Primer Sequence (5'→3')	Annealing temperature (ºC)	Product Size (bp)	Reference
23S rRNA *(H. pylori)*	F:5'AGGTTAAGAGGATGCGTCAGTC3'	57°C	267	[12]
	R:5'CGCATGATATTCCCATTAGCAGT3'			
cagA	F:5'AATACACCAACGCCTCCAAG3'	60°C	397	[13]
	R:5'TTGTTGCCGCTTTTGCTCTC3'			
sabA	F:5'TTTTTGTCAGCTACGCGTTC3'	56°C	622	[14]
	R:5'ACCGAAGTGATAACGGCTTG3'			

The PCR reaction was performed in a final volume of 25 µL, containing 12.5 µL of Master Mix 2X, 1 µL of each primer (10 pmol/μL), 4 µL of extracted DNA, and 8.5 µL of distilled water.

The *H. pylori* HC168 strain (cag A^+^/sab A^+^) from the Helicobacter Research Laboratory collection at the Research Institute for Gastroenterology and Liver Diseases, Shahid Beheshti University of Medical Sciences, Tehran, Iran, was used as the positive control for cagA and sabA, while the *H. pylori* J99 standard strain (CCUG471167) was used to validate the specificity and sensitivity of the primers. A no template control (NTC) reaction was included as the negative control.

PCR thermal cycling conditions

For the amplification of the 23S rRNA gene of *H. pylori*, the PCR cycle began with an initial denaturation at 95°C for five minutes, followed by 33 cycles of denaturation at 94°C for 45 seconds, primer annealing at 57°C for 45 seconds, and extension at 72°C for 45 seconds. A final extension was performed at 72°C for five minutes.

For the cagA gene, an initial denaturation was carried out at 95°C for five minutes, followed by 35 cycles of denaturation at 95°C for 30 seconds, annealing at 60°C for 35 seconds, and extension at 72°C for 35 seconds, with a final extension at 72°C for five minutes.

For the sabA gene, the thermal cycle started with an initial denaturation at 94°C for four minutes, followed by 33 cycles of denaturation at 94°C for 60 seconds, primer annealing at 56°C for 60 seconds, and extension at 72°C for 60 seconds. The final extension step was performed at 72°C for 10 minutes.

The PCR products were analyzed by gel electrophoresis using a 1% agarose gel and a 100 bp DNA Ladder (Yekta Tajhiz, Iran), and visualized under ultraviolet (UV) light. The presence of target bands indicated a positive result for *H. pylori* or its virulence genes.

Statistical analysis

Data were analyzed using IBM SPSS Statistics for Windows, Version 27 (Released 2020; IBM Corp., Armonk, New York, United States). The Chi-square test, Fisher’s exact test, independent t-test, and logistic regression were used to compare the prevalence of *H. pylori* between groups and to assess the correlation between oral hygiene factors and *H. pylori* detection. Agreement between the salivary PCR and gastric biopsy results was evaluated using Cohen’s Kappa statistic, and the Kappa coefficient and its significance were calculated in SPSS. A p-value <0.05 was considered statistically significant.

## Results

A total of 100 children participated in the study, comprising 45 boys (45%) and 55 girls (55%). The participants were aged four to 18 years, grouped in two-year intervals. The average DMFT/dmft score across all participants was 5.04 ± 3.42, with a minimum score of zero and a maximum score of 17. The majority of children, 83 (83%), ate fast food once a week or less, while eight (8%) ate it two to three times a week, and nine (9%) consumed it more than three times a week. Dairy consumption was reported by 60 children (60%) at a frequency of two to three times per week, while 51 (51%) ate vegetables more than three times a week. In contrast, 97 children (97%) consumed canned food once a week or less. Regarding dental visits, 45 children (45%) went to the dentist only when they had a problem, 42 (42%) visited annually, and 13 (13%) attended routine check-ups twice a year. In terms of oral hygiene, 63 children (63%) brushed their teeth once daily, 25 (25%) brushed two to three times a week, and 12 (12%) brushed less frequently. The educational levels of the parents were as follows: the majority, 37 fathers (37%) and 31 mothers (31%), had a high school diploma, while 30 fathers (30%) and 29 mothers (29%) held a bachelor's degree. The remaining parents had lower education levels, while only a small proportion had higher qualifications.

No significant correlation was found between gender and gastric biopsy results or between age and either gastric or salivary *H. pylori* contamination (p>0.05). However, a significant correlation was observed between gender and the presence of *H. pylori *in saliva p=0.013). Parental education levels were not significantly associated with either gastric or salivary *H. pylori* prevalence (p<0.05). Furthermore, while different food consumption patterns did not show a significant relationship with gastric *H. pylori* (p<0.05), dairy consumption was significantly associated with the prevalence of *H. pylori* in saliva (p=0.029).

Additionally, no significant correlation was found between the frequency of tooth brushing and the presence of *H. pylori* in the saliva (p<0.05). However, individuals who visited the dentist for regular check-ups (twice a year) were significantly less likely to have oral *H. pylori* infection compared to those who only visited when experiencing problems (Table 2).

**Table 2:** Correlation of demographic, oral hygiene, and dietary variables with gastric and salivary H. pylori infection in children

Variable	p-value	Significance	Odds Ratio (OR)
Gender and gastric biopsy	0.143	Not Significant	-
Gender and salivary *H. pylori*	0.013	Significant	-
Age and gastric biopsy	0.827	Not Significant	-
Age and salivary *H. pylori*	0.88	Not Significant	-
Father’s education and gastric biopsy	0.446	Not Significant	-
Father’s education and salivary *H. pylori*	0.456	Not Significant	-
Mother’s education and gastric biopsy	0.232	Not Significant	-
Mother’s education and salivary *H. pylori*	0.177	Not Significant	-
Fast Food consumption and gastric biopsy	>0.05	Not Significant	-
Dairy consumption and salivary *H. pylori*	0.029	Significant	-
Tooth brushing frequency and salivary *H. pylori*	0.175	Not Significant	-
Dental visit frequency and salivary *H. pylori*	-	Significant	14.6

**Figure 1:**
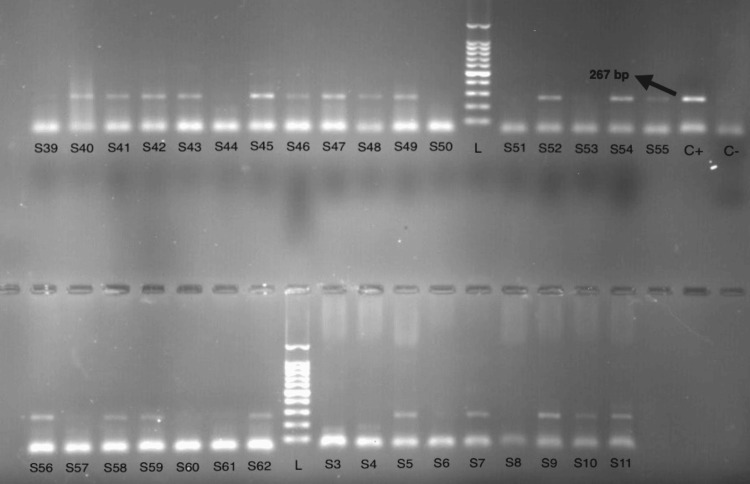
Electrophoresis results of the samples for the 23S rRNA gene L: 100 bp DNA ladder; S: saliva samples (S1, S2, S3…); C+: positive control; C−: negative control.

In this study, 53 children (53%) had saliva samples that tested positive for *H. pylori*, while 47 (47%) tested negative (Figure 1).

**Figure 2:**
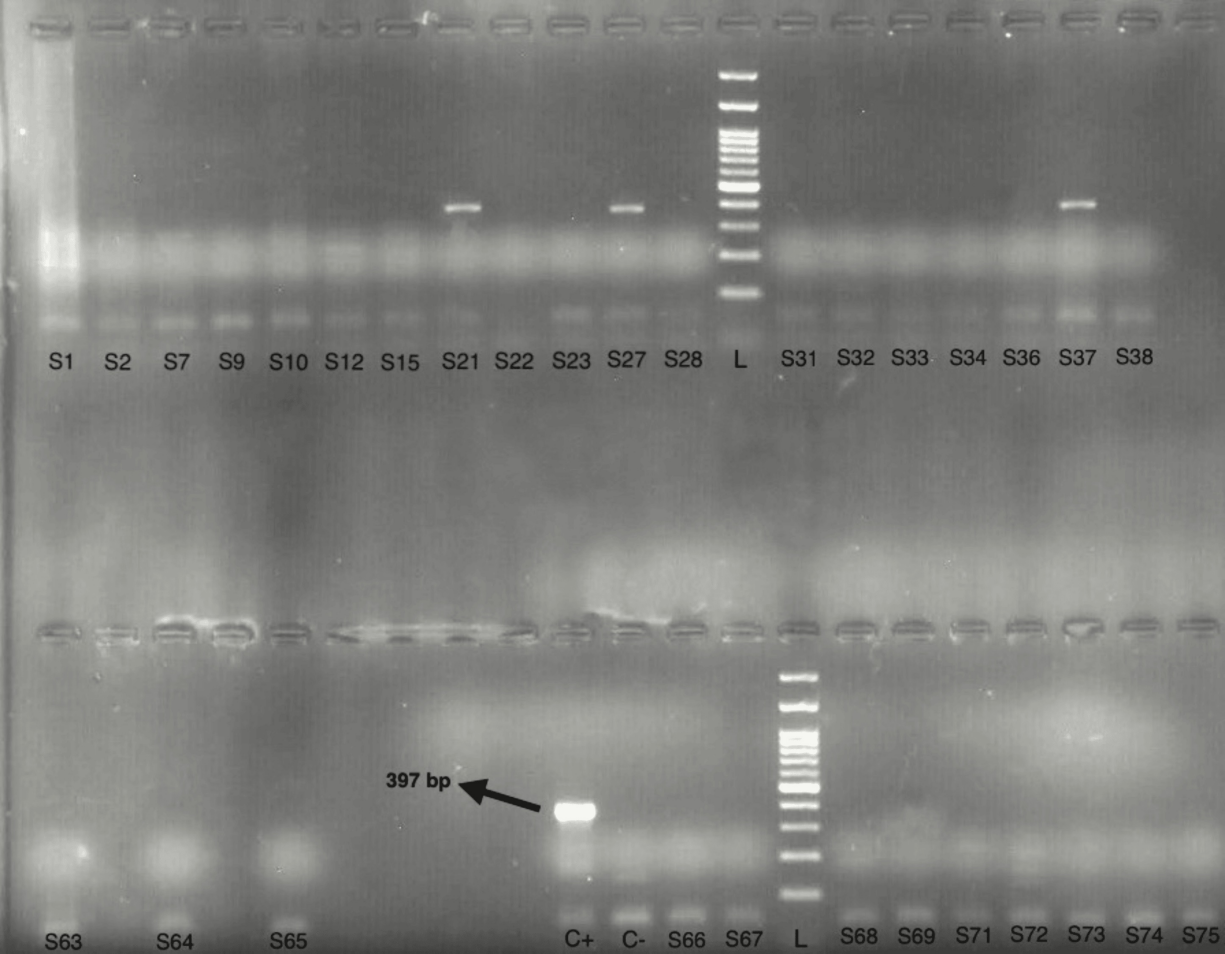
Electrophoresis results of the samples for the cagA gene L: 100 bp DNA ladder; S: saliva samples (S1, S2, S3…); C+: positive control; C−: negative control.

Among the saliva-positive samples, eight children (8%) carried the cagA gene (Figure 2), indicating a low prevalence of aggressive bacterial strains, and none of the samples tested positive for the sabA gene, suggesting the absence of highly virulent strains.

Similarly, gastric biopsy results showed that 61 children (61%) tested positive for *H. pylori*, while 39 (39%) tested negative.

This study found a significant association between gastric *H. pylori* infection and worse dental health. Children with gastric *H. pylori* infection had higher DMF/dmf scores and more decayed teeth than those with negative gastric biopsies (p<0.001). Similarly, salivary *H. pylori *infection was linked to increased dental decay and fillings, with the positive group showing significantly higher scores for both (p<0.001 for decay, p=0.029 for fillings). However, no significant association was found between *H. pylori* and tooth loss (p=0.202 for saliva, p=0.595 for gastric biopsy). These results emphasize the impact of *H. pylori* on dental decay and fillings, but not on missing teeth (Table 3).

**Table 3:** Prevalence and concordance of H. pylori in gastric biopsy and saliva samples with dental health indicators (DMF/dmf) DMF: Decay, Filling, Missing.

Dental health indicator	Gastric biopsy (Positive)	Gastric biopsy (Negative)	Saliva (Positive)	Saliva (Negative)	p-value
DMF/dmf	7.00 ± 2.79	3.62 ± 2.51	4.07 ± 2.18	3.74 ± 2.01	<0.001
Decay	4.21 ± 2.56	1.77 ± 1.74	4.45 ± 2.96	1.91 ± 1.76	<0.001
Filling	2.23 ± 1.56	1.41 ± 1.48	2.30 ± 1.82	1.47 ± 1.58	0.029
Missing	0.56 ± 1.46	0.44 ± 1.68	0.64 ± 1.87	0.36 ± 1.46	0.202

The relationship between gastric and salivary *H. pylori* presence was assessed to evaluate the sensitivity and specificity of salivary testing. The Kappa value of 0.838 indicated a high agreement between the two methods, suggesting a strong correlation. The sensitivity of the salivary test was 86.9%, and the specificity was 100%. This means that when *H. pylori* is absent from the stomach, the saliva test will correctly identify it 100% of the time, with no false positives. However, the test may miss some cases (false negatives), as seen by the sensitivity of 86.9%.

## Discussion

This study examined the prevalence of *H. pylori* in children's saliva and its association with dental health, particularly dental caries. Among the 100 children studied, 45% were male subjects and 55% female subjects, with no significant gender difference in gastric *H. pylori *infection (p=0.143). However, a significant gender difference in oral *H. pylori* prevalence was observed (p=0.013), with boys showing higher rates. This finding aligns with existing literature that has reported higher oral *H. pylori* prevalence among boys, likely due to differences in oral hygiene and behavior [15,16].

Age did not show a significant correlation with *H. pylori* infection (p>0.05), consistent with Nasrolahei et al. (2003) [17]. Nonetheless, research from other studies suggests that *H. pylori* prevalence tends to increase with age, likely due to prolonged exposure and a higher risk of transmission [5,18]. Parental education levels also did not significantly correlate with *H. pylori *prevalence (p>0.05), although descriptive data suggested a potential association between lower parental education and higher infection rates. However, this relationship is likely influenced by factors such as hygiene practices and access to healthcare, which have been highlighted as important contributors in the literature [5,16].

Dietary habits were also examined in relation to *H. pylori* prevalence. Regular consumption of dairy products, particularly fermented varieties rich in probiotics like *Lactobacillus* and *Bifidobacterium*, was found to significantly reduce the likelihood of *H. pylori* infection, aligning with findings from Keikha et al. (2024). Probiotics are thought to inhibit colonization through the production of antimicrobial metabolites and modulation of the immune response [19]. In contrast, the intake of fast foods, vegetables, and canned foods did not appear to have a significant impact on infection rates, supporting findings from a study that similarly failed to observe a strong connection between processed foods and *H. pylori* prevalence [10].

While frequency of brushing did not correlate significantly with *H. pylori* presence in the saliva (p=0.175), the study did reveal a notable finding regarding dental check-ups. Children who visited the dentist twice a year were found to be 14.6 times less likely to have oral *H. pylori* infection compared to those who only sought dental care when problems arose. This reinforces the importance of regular dental visits in maintaining oral health and reducing the microbial load in the mouth, ultimately helping to prevent *H. pylori* colonization [14].

Furthermore, analysis of the saliva samples revealed that 53 children (53%) were positive for the *H. pylori* 23S rRNA gene, a marker of the bacterium’s presence. However, only eight children (8%) tested positive for the cagA gene, suggesting that most oral strains of *H. pylori *lacked the aggressive virulence factors associated with systemic disease. Additionally, none of the samples were positive for the sabA, further indicating that the strains in the oral cavity might be less virulent. These results suggest that the oral cavity may act more as a passive reservoir for *H. pylori* rather than a primary site of its pathogenic effects, as supported by previous studies [20,21]. However, given the limited number of virulence genes analyzed and the relatively small sample size, this conclusion should be interpreted with caution.

A key finding of this study was a significant association between gastric *H. pylori* infection and dental caries, as evidenced by higher DMFT/dmft scores (p<0.05), indicating that children with gastric *H. pylori* infection had more severe dental caries. This result aligns with Iwai et al. (2022), who reported that individuals with gastric *H. pylori* infection had significantly more decayed teeth, suggesting that *H. pylori *may contribute to the exacerbation of dental caries [22]. Similarly, oral *H. pylori* infection was significantly associated with higher DMFT/dmft scores (p<0.05), suggesting that the presence of *H. pylori* in the oral cavity may play a role in the development of dental decay. Previous studies have also demonstrated that children with oral *H. pylori *tend to have more decayed teeth and a higher need for dental fillings [7,23]. These findings suggest that oral *H. pylori* may not only contribute to the persistence of gastric infection but also worsen the oral microbiome, creating a more favorable environment for cariogenic bacteria such as *Streptococcus mutans*.

The study also found no significant correlation between the "Missing" component of the DMFT index and *H. pylori* infection (p=0.973), reinforcing the notion that *H. pylori* is more closely associated with active decay and fillings rather than tooth loss. This is likely because tooth loss is influenced by other factors, such as orthodontic treatment or extractions, which are not directly related to *H. pylori* infection [7].

Finally, a strong correlation was observed between gastric biopsy and salivary PCR results, with a Kappa value of 0.838, indicating high agreement between the two methods. Salivary PCR demonstrated a sensitivity of 86.9% and a specificity of 100%, making it a reliable, non-invasive method for screening *H. pylori *infection in children. These results are consistent with previous studies that have highlighted the high sensitivity and specificity of salivary PCR as an alternative to gastric biopsy, further supporting its use as a non-invasive diagnostic method [20,24].

While these findings are promising, some limitations must be noted. The sample size was relatively small, and although statistically significant results were observed, the findings may not be fully generalizable to larger populations. Additionally, this study was conducted at a single center, with participants referred for endoscopy, which may not represent the broader pediatric population. Furthermore, the cross-sectional design of the study means that causal relationships cannot be inferred. Longitudinal studies are needed to explore causality and the impact of *H. pylori* eradication on oral health outcomes.

## Conclusions

This study highlights the potential role of saliva as a reservoir for *H. pylori* in children, associating its presence with increased dental caries, particularly active decay and fillings. The strong correlation between salivary PCR and gastric biopsy suggests that salivary PCR could be an effective, non-invasive method for early detection of *H. pylori *infection. While oral *H. pylori *strains are predominantly nonvirulent, they may contribute to the transmission of infection. These findings underscore the need for further research with larger sample sizes from diverse regions of Iran to better assess these results, explore additional virulence factors, and gain a deeper understanding of the impact of *H. pylori* on oral health. Longitudinal studies and interventional trials are necessary to establish causality and evaluate the effects of *H. pylori *eradication on oral health outcomes.
